# Economic Impact of Infected Total Knee Arthroplasty

**DOI:** 10.1100/2012/196515

**Published:** 2012-04-19

**Authors:** Maximilian Haenle, Christina Skripitz, Wolfram Mittelmeier, Ralf Skripitz

**Affiliations:** ^1^Departement for Orthopaedic Surgery, University of Rostock, Doberaner Stra*β*e 142, 18057 Rostock, Germany; ^2^Institute for Medical Microbiology, Virology and Hygiene, University of Rostock, Schillingallee 70, 18057 Rostock, Germany

## Abstract

*Background.* An enormous economic impact can be observed for infected total knee arthroplasties (TKA). The aim of the present study was to evaluate whether a cost covering treatment of infected TKA is feasible in the German DRG System. *Patients and Methods.* Average total treatment costs were evaluated for infected TKA and compared with a matched pair of primary TKA. Data was generated using the health record and the hospitals' health information system. Results were evaluated and compared regarding the total personnel and material costs with respect to the financial receipts. *Results.* A total of 28 patients diagnosed with an infected TKA were included. A significant increase in the average length of stay, use of medical supplies and third party medical examinations were found for the infected TKA. An average deficiency of 6,356€ per patient was observed for the infected TKA. An average profit of 927€ per patient was made performing primary TKA. *Conclusions.* A cost-effective treatment of infected TKA was not feasible with the receipts from the German DRG System. An adaption of the receipts has to be evaluated. Moreover, other measures have to be considered in order to achieve a comprehensive medical yet financial reasonable standard in the treatment of infected TKA and THA.

## 1. Introduction

### 1.1. Infected Total Knee Arthroplasties

Total knee arthroplasties (TKAs) helped to improve the quality of life of numerous patients and are considered successful standard operations in orthopaedic surgery. Annual implant rates add up to 148.000 TKA in Germany alone [1]. Despite advances in operative techniques and environments, peri-prosthetic infections remain feared and severe complications after TKA. Deep infection accounts for roughly 20% [2] of TKA revision operations. The reported incidence lies between 0.5 and 1% [3, 4]. A number of risk factors for deep infection after TKA, such as male sex, rheumatoid arthritis (RA), an American Society of Anaesthesiologists (ASA) risk score >2, diabetes mellitus, and morbid obesity, have been identified [5]. The most common type of infections is early and delayed TKA infections [4]. Again the most frequently isolated pathogens are *Staphylococcus aureus* and coagulase-negative staphylococci, which account for up to 58% of periprosthetic TKA infections [4]. Beside the devastating consequences for the patient, an enormous economic impact upon the treating hospital can be observed.

### 1.2. Economic Impact of Infected Total Joint Arthroplasties

Additional average treatment costs of a single infected total joint prosthesis for both medical and surgical treatment are estimated to be $30,000 and more [6]. In total, roughly $1.8 billion additional costs are caused by infected joint prosthesis and fracture fixation devices per year in the United States of America (USA) [6]. The treatment costs of infected total joint prostheses hence pose a vast burden directly to the hospital and consequently the corresponding health care system. Beside the financial impact, the enormous psychosocial consequences should of course not be neglected. Nevertheless treatment of infected TKA becomes more and more of an economic issue as certain therapy costs are not or insufficiently reflected within the German Diagnosis-Related Groups (G-DRGs) [7].

### 1.3. DRG System

The DRG System was originally developed in the 1970s as a patient classification system. Patients were grouped into a limited number of distinct medical diagnostic categories in order to measure each individual patient's consumption of hospital resources [8]. The primary purpose of the DRGs was to serve as a basis for quality assessment, to improve care for patients and for the better use of limited and expensive medical resources [8, 9]. It only later evolved to be an efficient measure of cost control [8]. It is nowadays regarded as a fixed rate reimbursement system in order to avoid an implausible variation of costs [10]. Within the system each individual case is assigned to a corresponding DRG. Complications or comorbidities may increase the treatment costs and consequently the reimbursement. The severity of a disease can then be recognized and accounted for within the DRGs. The G-DRGs were compulsory introduced on January 1, 2004, based upon the Australian-Refiened DRGs. They are designed to be evolving due to an annual revision and recalculation with respect to medical cost and performance. Regarding TKA within the G-DRGs, the annual restructuring led to a subsequent improvement of the differentiation criteria [11, 12]. Furthermore, the introduction of a distinct OPS-Code for the surgical treatment of severe infections leads to an improved reflection of multiple, complex procedures [11] that previously had only insufficiently been reimbursed.

### 1.4. Aims and Scope

In the present study, a retrospective analysis of total treatment costs of infected TKA was performed and compared to a case control group of noninfected primary TKA. The aim of the present retrospective study was to evaluate whether a cost-covering treatment of infected TKA is feasible in the German DRG System. Moreover, the range of profit respectively loss will be demonstrated.

## 2. Patients and Methods

Data was retrospectively analyzed from January 2004 until December 2007. Initially every infected TKA, treated at the Department of Orthopaedic Surgery of the University Hospital Rostock (Orthopädische Universitätsklinik Rostock, OUK), was detected. This included patients who did receive a primary or revision TKA in our department as well as patients primarily treated in other hospitals and transferred to our institution. The case control group consisted of 21 patients who received a primary TKA in our department during the analysis period. Case control patients were selected in a blinded manner. Definition of the case control group sample size is based upon calculations of the Institute of Biostatistics and Informatics in Medicine and Ageing Research, University of Rostock. Exclusion criteria of the case control group were metabolic bone diseases, malign bony tumours, prior radiotherapy, prior ipsilateral total joint arthroplasty within the last 12 months, or prior contralateral total joint arthroplasty within the last 6 months. First of all, infected TKAs were classified with respect to the detected pathogens. Regarding the timing of infection, infected TKAs were differentiated in early (<2 months after implantation), delayed (>2 months until 1 year after implantation), and late infections (>1 year post implantation). Data was systematically generated using the health record and the hospitals' health information system (SAP, Walldorf, Germany). Beside the clinical parameters, DRG relevant costs and performance variables were evaluated. This included length of stay, principal and secondary diagnoses, length, number, and type of operation procedures, number of hospital stays, length of anaesthesiology, microbiological and histological exams, laboratory costs, blood products, and consults. Based upon the federal employee tariff (“Bundes Angestellten Tarif”, BAT), which was effective during most of the period of analysis, calculation of medical personnel costs was performed. Hereby, the official standard weekly working time of 38.5 hours was assumed. Analogous, nonmedical personnel costs were calculated using the KR V and VI. To simplify matters, vacation, sickness, age, and marital status were left unconsidered. According to the Central Controlling Unit of the University Hospital Rostock (CCU), costs per anaesthesia minute add up to 7.30€. This includes costs for medical and nonmedical personnel as well as supply materials. The average daily costs per patient treated in an Intensive Care Unit (ICU) are stated to be 1,265€ [13] and were used accordingly. Costs for medical supplies and pharmaceuticals were calculated after analyses of the health record, anaesthesiology protocols, surgical reports, and surgical protocols. Analogous, total costs of third party medical services, namely, microbiological, histological, laboratory, and radiological examinations were evaluated. A total of 21.23€ was charged per microbiological investigation for performing a fungal analysis, an analysis in CO_2_-atmosphere, an anaerobic and an aerobic analysis, as well as a sensibility testing in the break-point method. According to the CCU, a fixed rate of 24.05€ per histological examination was accounted. Furthermore the CCU supplied detailed costs for individual radiological and laboratory examinations. Homologous erythrocyte concentrates (ECs) amount to 80.46€, and autologous EC to 77.18€. Calculation of the exact financial reimbursement was performed using the G-DRG-Online Grouper with respect to the primary and secondary diagnosis as well as performed procedures, age, sex, and length of hospitalisation. The base value was set to be 2,964€.

Statistical evaluation was performed using the Mann-Whitney test for nonparametric independent samples with *P* < 0.05 considered to be significant.

## 3. Results

### 3.1. Patient-Related Results of Infected TKA

During the period of analysis of this retrospective study, a total of 55 infected TKAs were treated at our institution. Amongst these patients 22 were initially operated at the OUK, the remaining 33 transferred from other hospitals. However patients with a diagnosed TKA infection and only treated with a single debridement and lavage were not considered for this study. As a result, a total of 28 patients (8 males; 20 females; average age: 71.7 years) were included. The average length of hospitalisation was 48.2 days. Treatment on the ICU of the 28 patients was inevitable on average for 2.5 days. 10 patients were diagnosed with an early, 5 with a delayed, and finally 14 with a late TKA infection. *Staphylococcus epidermidis *was the most frequently isolated pathogen, followed by *Staphylococcus aureus*. Histological signs of infection were detected in 85.7% of the cases (*n* = 24). A total of 114 operations were performed on the 28 patients, which represents an average of 4.1 operations per patient. A two-stage TKA reimplantation was performed in 14 cases, a single-stage revision in another four cases. Out of the 28 patients, 19 received a blood transfusion due to anaemia. In average, these 19 patients received 7.2 EC. This again represents an average of 4.5 EC per patient with an infected TKA. The mean time of anaesthesia was found to be 95 minutes with a mean operation time of 64 minutes.

### 3.2. Cost-Specific Results of Infected TKA

**Figure 1:**
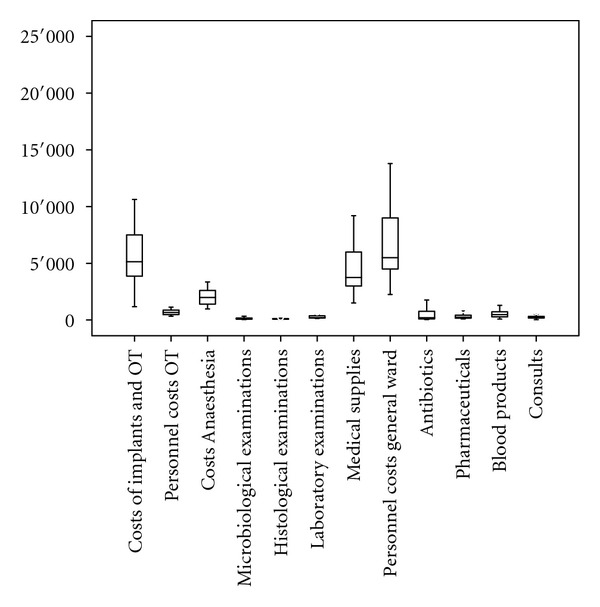
Mean, standard deviation, minimum, and maximum values of the various positions for infected TKA in Euro.

The average total treatment costs were 25,194€ per patient for the infected TKA (Table 1; Figure 1). 44.2% of the total costs were allotted to personnel and 55.8% to supply materials. Within the operation unit, an average of 8,7334€ expenses was observed. Thereby personnel costs were 2,842€ and costs for medical supplies were 5,892€. Calculated cumulative costs from the ICU were 3,082€, with personnel costs of 1,541€ and 1,541€ costs for medical supplies. The average costs on the general ward were found to be 13,379€, in detail 6,760€ personnel costs and 6,618€ charges for medical supplies (Table 1).

### 3.3. Patient-Related Results of Case Control Group

The case control group of primary noninfected TKA consisted of 21 patients (10 males; 11 females; average age: 65.6 years). The average length of hospitalisation was 13.4 days. Treatment on the ICU was unnecessary for any patient of the case control group. As a routine procedure, in 13 patients an intraoperative microbial swab was taken. Furthermore tissue was sent for histological examination. Neither histological signs of infection nor a positive microbial culture was hereby detected. Out of the 21 patients, 6 received a blood transfusion due to anaemia. In average, these 6 patients received 2.2 EC. This again represents an average of 0.6 EC per patient of the case control group. The mean time of anaesthesia was found to be 112 minutes with a mean operation time of 82 minutes.

### 3.4. Cost-Specific Results of Case Control Group

The average total treatment costs were 6,889€ per patient for the case control group (Table 2). 43.4% of the total costs were allotted to personnel and 56.6% to supply materials. Within the operation unit, an average charge of 3,266€ was observed. Thereby personnel costs accumulated to 1,091€ and costs for medical supplies to 2,174€. Total cumulative costs on the general ward were found to be 3,623€, in detail 1,896€ personnel costs and 1,728€ costs for medical supplies (Table 2).

### 3.5. Cost Analysis

Regarding the 28 patients with the diagnosis of an infected TKA, a mean loss of 6,356€ between reimbursements from the G-DRGs and total calculated costs was observed per patient. This results in a cost coverage of only 74.77%. On the other hand, a mean profit of 927€ can be achieved performing primary TKA. The cost coverage is calculated to be 111.86% with an average reimbursement of 7,816€ and total costs of 6,889€ (Table 3).

## 4. Discussion

In the present study, a cost analysis was performed regarding 28 patients diagnosed with an infected TKA, treated at the OUK. The corresponding costs were compared to a case control group of 21 patients with a noninfected primary TKA. We were able to demonstrate that a profit of 927€ can be achieved performing primary TKA, where on the other hand a mean loss of 6,356€ was observed treating infected TKA (Table 3). Breaking down the matters of expense, obvious differences between length of hospitalisation and cumulative operation time can be found for infected and noninfected TKA. This again is represented by the considerable increase of total costs. The prolonged length of hospitalisation and cumulative operation time account for the increase of personnel costs within the operation unit and on the general ward. Furthermore, the use of antibiotics as well as other pharmaceuticals and erythrocyte concentrates is significantly increased for patients with an infected TKA (*P* < 0.05). The same could be demonstrated for costs arising from third party medical services and medical supplies. Finally, treatment costs from the ICU were only observed for patients with infected TKA (Tables 1 and 2).

In literature implant costs for primary TKA have been found to be $10,989 in the USA in the late 1980s and accounted for roughly 40% of the total charges [14]. In the present study, we were still able to demonstrate similar charges regarding the cost analysis of primary, noninfected TKA nowadays in Germany. However total billed surgical fee by the surgeon alone accounted for 20% of the total charges [14] whereas in the present study the majority of costs are due to the sum of charges of cumulative operation time, implants and pharmaceuticals. This might partly be due to the different accounting procedures in the USA and Germany. Recently, the hospital economics of primary TKA have been analyzed [15]. From 1991 to 2009 a loss per case of US $2,172 was converted into a profit of US $2,086 per primary TKA. According to the authors, reduction of hospital length of stay and reduction of knee implant costs were the major drivers of the hospital expense reduction. In 1991, the average duration of hospital stay was 9.0 days whereas, in 2008, it was reduced to an average of only 3.7 days [15]. According to our results, a profit may also be achieved performing primary TKA in the G-DRG System. However our own results show a considerably higher duration of hospital stay of 13 days, what explains the lower profit margin which might therefore be increased by adapting to international standards of hospitalisation. In 2008, hospital expenses in primary TKA was $11,002 in the USA [15]. We were able to demonstrate similar expenses of 6,889€ between 2004 and 2007. As opposed to our results, costs for pharmaceuticals, laboratory exams, and operation time were found to be higher in the USA. However, regarding the percentage they seem to be more or less consistent with our results from a German University Hospital [15]. Comparable expenses of US $7,331 per primary TKA can furthermore be found in Canada (charges converted into US dollars) [16]. The authors furthermore demonstrated that $1,667 have to be spent for every 10-point increase of the WOMAC (Western Ontario McMaster Arthritis Questionnaire) [17] for a patient undergoing primary TKA compared to $2,602 per 10-point increase for patients undergoing aseptic TKA revision [16]. No such calculation has however been performed for the treatment of infected TKA. Nevertheless surgical treatment of an infected TKA requires three to four times the resources of hospital and surgeon compared with a primary TKA and approximately twice the resources of a nonseptic revision TKA [18]. A net loss of approximately $30,000 (22,545€) was hereby observed per Medicare patient [18]. Yet almost two decades of age and from the USA, these results are still consistent with our present findings. It has been stated that the reimbursement rates will not even cover hospital costs for TKA revision surgery with substantially higher costs for patients with deep joint infections. It is even suggested that TKA revision due to deep infection is one of the highest resource consumption procedures in orthopaedic surgery [19]. This again is supported by the recent findings of a significant increase of mean treatment costs for infected TKA compared to noninfected revision TKA [20]. A significant increased length of stay and mean costs for examinations is demonstrated for the treatment-infected TKA [20]. These results again coincide with our findings. However in our study we furthermore show an increase in implant costs and the financial impact of the necessity due to intensive care treatment.

In summary, there is a consensus in the present literature that a cost covering treatment of infected TKA is not feasible in most healthcare systems. A profit however may be achieved performing primary TKA. Nevertheless there is a lack of detailed cost coverage calculations regarding the treatment of infected TKA. Our results therefore complement the present literature by the point of view from the G-DRG System.

Next to the general limitations of a retrospective study, there are further obvious limitations. The present paper only focuses on direct medical costs whereas other, nonmedical costs such as loss of production, salary, and social security taxes were not included. The socioeconomic impact of infected TKA is therefore left unconsidered. Furthermore, the data was only generated at one institution. Despite the fact that this offers the opportunity of a detailed and patient-specific analysis of clinical data and charges, only a limited conclusion may be drawn upon the general German healthcare system. Further studies are anticipated in order to compare our results with the results from other German hospitals. Moreover, prices were evaluated in 2011 and no adjustments of inflation have been taken into account. Another limitation is of course that only infected TKAs were analysed. A comparison between noninfected and infected TKA revision is hence not possible in the present study. We furthermore abdicated on the differentiation between infected primary and revision TKA, as, apart from a higher rate of infection after revision TKA, no significant differences in the general treatment and hence charges are expected. Finally comparability of our results with results from other publications is hindered due to the shortened length of stay internationally.

## 5. Conclusion

During the observed period, a cost-covering treatment of infected total knee arthroplasties was not feasible with the reimbursements from the German DRG System. The average loss per patient was 6.356€. The main reasons were found to be the significant increase of personnel and supply material costs in comparison to the noninfected primary total knee arthroplasties (*P* < 0.05). Besides, the necessary treatment procedures are not sufficiently reflected and rewarded within the German DRG System. An adaption of the reimbursements has hence to be evaluated. However, other measures have to be considered in order to achieve cost-effectiveness. Multicentre cost analysis and extensive measures of quality control are anticipated, in order to achieve a comprehensive medical yet financial reasonable standard in the treatment of infected total knee arthroplasties.

## Figures and Tables

**Table 1:** Mean absolute und percentage costs of relevant positions in infected TKA.

Cost of implants	5.892,19€	23,39%
Laboratory examinations	268,32€	1,07%
Microbiological examinations	132,65€	0,53%
Histological examinations	111,66€	0,44%
Radiological examinations	247,83€	0,98%
Blood products	502,99€	2,0%
Antibiotics	518,64€	2,05%
Pharmaceuticals	316,5€	1,26%
Medical supplies	4.520,57€	17,94%
Personnel costs OT	714,08€	2,83%
Costs Anaesthesia	2.127,69€	8,45%
Personnel costs general ward	6.760,47€	26,83%
Costs ICU	3.081,61€	12,23%

Total costs	25.194,3€	100%

OT: Operation Theatre; ICU: Intensive Care Unit.

**Table 2:** Mean absolute and percentage costs of relevant positions case control group (noninfected primary TKA).

Cost of implants	2.174,48€	31,57%
Laboratory examinations	88,13€	1,28%
Microbiological examinations	15,16€	0,22%
Histological examinations	4,58€	0,07%
Radiological examinations	169,34€	2,46%
Blood products	42,15€	0,61%
Antibiotics	5,66€	0,08%
Pharmaceuticals	96,82€	1,41%
Medical supplies	1.305,86€	18,96%
Personnel costs OT	274,17€	3,98%
Costs Anaesthesia	816,90€	11,86%
Personnel costs general ward	1.895,60€	27,52%

Total costs	6.888,85€	100%

OT: Operation Theatre.

**Table 3:** Cost analysis of infected TKA and the case control group with reimbursements from the G-DRGs in Euro (€).

Group	Number of patients	Mean cost per patient	Mean reimbursement (G-DRGs)	Mean profit/loss	Cost coverage
Infected TKA	28	25,194€	18,838€	−6,356€	74.77%
Case control group	21	6,889€	7,816€	+927€	111.86%
